# Hospital-free days in the first year after lung transplantation and subsequent survival

**DOI:** 10.1016/j.jhlto.2024.100127

**Published:** 2024-07-18

**Authors:** Reda E. Girgis, Austin Frisch, Cameron K. Lawson, Benjamin Kowalske, Lindsey LeQuia, Ryan J. Hadley, Sheila Krishnan, Gayathri Sathiyamoorthy, Edward T. Murphy

**Affiliations:** Richard DeVos Heart and Lung Transplant Program, Corewell Health and Michigan State University College of Human Medicine, Grand Rapids, Michigan

**Keywords:** hospital-free days, survival, lung transplant, readmissions, length of hospital stay

## Abstract

**Background:**

Complications occurring during the first postoperative year after lung transplantation increase the risk of long-term mortality. These events often lead to prolonged and repeated hospitalizations. We sought to assess the relationship between days outside the hospital or hospital-free days (HFD) during the first post-transplant year among 1-year survivors and subsequent retransplant-free survival.

**Methods:**

In a single-center study, we derived total inpatient days (initial transplant episode, readmission, and emergency room/observation) from the electronic medical record of lung transplant recipients who survived 1 year. The cohort was divided into HFD quartiles and Kaplan-Meier curves of subsequent transplant-free survival were compared with log-rank analysis. A Cox proportional hazards model was used to test the association of HFD with outcome and adjusted for selected variables.

**Results:**

Among 238 patients, 42 deaths and 2 retransplants occurred after a median of 3.6 years post-transplant. The median HFD was 341 (interquartile range: 324, 348). Estimated transplant-free survival at 3 and 5 years post-transplant in the lowest quartile of HFD (79% and 56%, respectively) was considerably worse compared with the first quartile (98% and 94%; *p* < 0.01). Fewer HFD were associated with subsequent death or retransplant [hazard ratio: 0.90 (95% confidence interval: 0.85, 0.94; *p* < 0.001) for each 10-day increase] and remained significant after adjusting for several potentially confounding variables.

**Conclusions:**

HFD during the first year after lung transplantation is a predictor of subsequent long-term outcomes and may be a useful surrogate marker for clinical trials during the early postoperative period.

## Background

Lung transplantation is an important therapeutic option for patients with advanced lung disease, yet long-term outcomes have remained stagnant in recent years with 5-year survival in the U.S. around 60%.^1^ Complications during the early postoperative phase, such as primary graft dysfunction (PGD), acute kidney injury (AKI), severe infections, and acute rejection, are important determinants of subsequent long-term survival. These events are expected to result in an increased need for hospitalization. Both longer duration of transplant episode length of stay^2^, ^3^ and readmissions^4^, ^5^, ^6^ have been associated with reduced long-term survival. The extent to which the total duration of hospitalization early post-lung transplant is associated with subsequent outcomes has not been investigated.

Hospital-free days (HFD) is a novel end-point that has been increasingly utilized in trials of critically and seriously ill populations that is both patient-centered and pragmatic.^7^ This outcome was recently proposed as an end-point for a clinical trial in lung transplant recipients comparing routine surveillance bronchoscopy with biopsy vs a donor-derived, cell-free DNA–guided strategy.^8^ The purpose of this study was to assess the relationship between HFD in the first-year post-transplant and subsequent transplant-free survival. We hypothesized that early post-transplant complications and other factors that contribute to long-term mortality and graft loss would be reflected in a reduced number of HFDs. If validated as a surrogate marker of survival, it may become a useful outcome measure for intervention trials during the early phase of lung transplantation.

## Materials and methods

### Patient cohort and study design

Lung transplant recipients who survived at least 1 year at our institution from February 2013 to August 2021 were included in this retrospective cohort study. Retransplants and multiorgan transplants were excluded. The primary objective was to assess the relationship between HFD in the first year and subsequent transplant-free survival, adjusted for other selected variables known to be associated with long-term outcomes. Determinants of HFD in the first year were also investigated. Guidelines from the Strengthening the Reporting of Observational Studies in Epidemiology Statement were followed.^9^

### Data collection

HFDs were defined as out-of-acute care hospitals, long-term acute care hospitals, emergency departments, or observation units and did not include acute or subacute rehabilitation units or home care.^7^ The definition in outcome studies also requires being alive, but we only selected 1-year survivors. Days in the hospital during the first post-transplant year were extracted from the electronic medical record and reflected the transplant episode length of stay, inpatient readmissions, and emergency room/observation unit days. Causes of readmissions were categorized based on discharge diagnoses. Hospital days that occurred outside our institution were not captured. The total of hospital days was subtracted from 365 to obtain HFD.

Selected recipient characteristics and perioperative variables were collected from our prospective lung transplant REDCap registry. Lung allocation score (LAS) was recorded at the time of transplant.^10^ Listing diagnosis was categorized according to LAS group as obstructive (A), pulmonary vascular (B), cystic fibrosis (C), and restrictive lung disease (D). The estimated glomerular filtration rate (eGFR) was calculated with the chronic kidney disease epidemiology collaborative equation.^11^ PGD was defined and graded according to International society for heart and lung transplantation consensus at 72 hours post-transplant.^12^ AKI within 7 days of transplant was defined and staged according to the kidney disease improving global outcomes criteria.^13^ The episodes of acute cellular rejection were defined and graded based on transbronchial biopsy findings.^14^ These were routinely performed for surveillance at 1, 3, 6, 9, 12, and 18 months post-transplant, as well as for clinical indications. The sum of all “A” grades was divided by the number of biopsies obtained during the first year to derive an acute rejection score (ARS). Grade B scores were not considered. Antibody-mediated rejection (AMR) in the first year was defined according to consensus guidelines as possible, probable, or definite^15^ and required therapeutic intervention. Chronic lung allograft dysfunction (CLAD) was defined according to consensus criteria.^16^ Subjects were followed until the time of death, retransplant, or end of the study observation period, August 2022. Causes of death were recorded and categorized. Recipients were managed according to our institutional guidelines with basiliximab induction, triple maintenance immunosuppression, and antimicrobial prophylaxis as previously described.^17^ This study was approved by our local institutional review board and written informed consent was obtained from all subjects as part of an ongoing registry.

### Statistical analysis

Numerical variables are presented as median (interquartile range, IQR) or mean (standard deviation) based on normality testing with Shapiro-Wilk test. Categorical variables are presented as counts (percentages). The cohort was divided into quartiles of HFD and Kaplan-Meier curves for survival beyond 1 year compared with log-rank analysis. *p* values for pairwise comparisons were adjusted with the Bonferroni-Holm method. The relationship between HFD during the first year and subsequent time to death or retransplant was assessed with a Cox proportional hazards model and adjusted for selected variables known or suspected to be associated with decreased long-term survival. Proportional hazard assumptions were checked with the test of proportionality. Linear regression was used to investigate the univariate association between selected variables and HFD in the first year. These variables were chosen based on clinical reasoning and literature as factors that could require or prolong hospitalization, as well as their availability in our registry. There was no missing data. Statistical analyses were done using R version 4.1.2 (January, 2021) with R Core Team (2021) (Vienna, Austria. URL https://www.R-project.org/).

## Results

### Patient cohort and outcomes

During the study period, 250 patients underwent a first-time lung transplant. Two combined heart-lung recipients and 10 subjects who died or were retransplanted within 1 year were excluded.

The demographic and clinical characteristics of the final cohort of 238 patients are listed in Table 1. During a median follow-up time of 1202 days (IQR: 828,1810), 42 deaths and two retransplants occurred at a median of 1307 days (IQR: 809, 1634). Kaplan-Meier transplant-free survival estimates at 3- and 5-years post-transplant were 90% and 75%, respectively. The causes of death are listed in Table 2. The leading cause (41%) was complications related to CLAD followed by malignancy (21%). Both retransplants were for CLAD. At the last follow-up, 45 subjects (19%) had developed CLAD at a median post-transplant interval of 771 Days (IQR: 586,1211).

**Table 1:** Cohort Characteristics

1-year survivors, N = 238	
Age at time of transplant, median (IQR)	65.0 (59; 69)
Male gender^a^	137 (58)
Primary diagnosis group	
A	77 (32)
B	8 (3.4)
C	14 (6)
D	139 (58)
Bilateral transplant	176 (74)
LAS at transplant, median (IQR)	39.3 (35.2; 47.5)
Ventilator and/or ECMO use pretransplant	14 (6)
Extracorporeal support used for transplant surgery^b^	111 (47)
Diabetes pretransplant	40 (17)
eGFR, median (IQR)	97 (88; 103)
CMV donor positive/recipient negative	50 (21)
Primary graft dysfunction grade at 72 hours	
0	127 (53)
1	60 (25)
2	34 (14)
3	17 (7)
Highest acute kidney injury stage by day 7	
0	145 (61)
1	57 (34)
2	19 (8)
3	17 (7)
Acute rejection score, median (IQR)	0.17 (0; 0.33)
Antibody-mediated rejection	20 (8.4)

Abbreviations: CMV, cytomegalovirus; ECMO, extracorporeal membrane oxygenation; eGFR, estimated glomerular filtration rate; IQR, interquartile range; LAS, lung allocation score.

a Values presented as N (%) unless otherwise indicated.

b Extracorporeal support refers to the use of ECMO or cardiopulmonary bypass for the transplant surgery.

**Table 2:** Causes of Death

Cause of death	Number of subjects
Chronic lung allograft dysfunction	17
COVID-19 related	4
Acute lung injury, unspecified	2
Antibody-mediated rejection	1
Refractory bronchial stenoses	1
Malignancy	9
Renal failure	1
Hepatic failure	1
Acute pulmonary embolism	2
Cardiovascular	2
Suicide	1
Unknown	1
Total	42

### Hospital-free days and readmission

**Figure 1:**
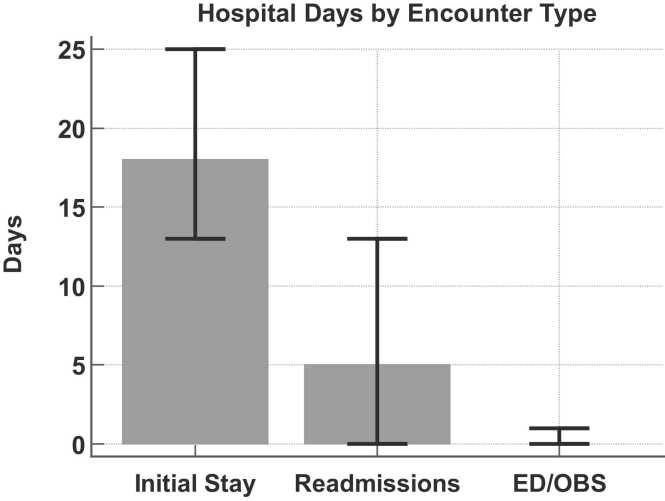
Bar graph demonstrating median hospital days (error bars: 25th-75th percentile) during the first post-transplant year for each encounter type: initial transplant episode, readmissions, and emergency department/observation days. N = 238. ED/OBS, Emergency department/Observation unit days.

The median number of HFDs in the first year for the cohort was 341 (IQR: 324, 348; range: 52-359). Figure 1 shows the hospital days by type of encounter. There were 369 readmissions in 161 (68%) patients. The median number of readmissions for this group was 2 (IQR: 1, 3; range: 1-10). The reasons for readmission are summarized in Table 3. Infection was the most common, followed by gastrointestinal ailments, airway complications, pleural disease, and cardiovascular events. Together, these 5 categories accounted for 69% of readmissions. Within the infection category, the lower respiratory tract represented the most common site (61%) and viruses were the identified organism in 63% of these infections.

**Table 3:** Causes of Readmission

Primary diagnosis	Count (%)
Infection	70 (19)
Airway complication	57 (15)
Gastrointestinal	57 (15)
Cardiovascular	35 (10)
Pleural complication	35 (10)
Renal	20 (5)
Musculoskeletal	12 (3)
Thromboembolism	11 (3)
Diabetes related	11 (3)
Neurological	6 (2)
Rejection	5 (1)
Other	50 (14)
Total	369

### Relationship between HFD and transplant-free survival

**Figure 2:**
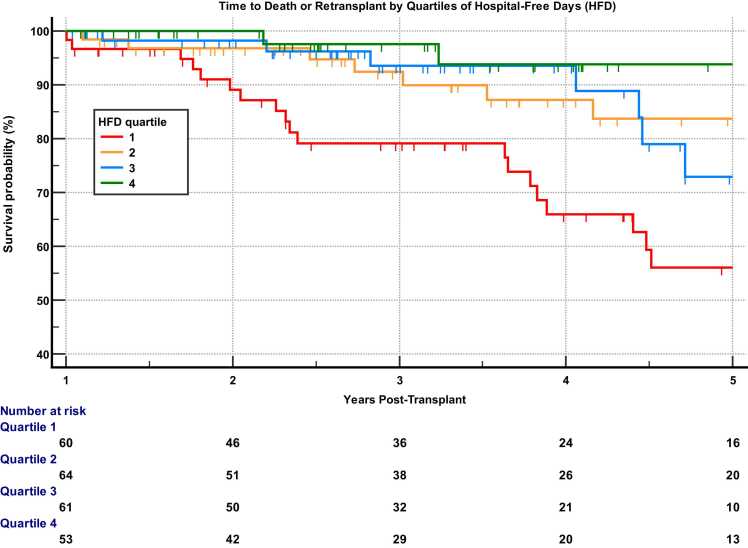
Kaplan-Meier curves demonstrating transplant-free survival in 1-year post-transplant survivors divided by quartiles of hospital-free days in the first year. Number of patients in each quartile is different due to ties in the number of hospital-free days at the cutoffs for quartiles. Ticks represent censored data. Overall log-rank *p* value = 0.002. Pairwise *p* values with Bonferroni-Holm adjustment for multiple comparisons: Q 1 vs 4: 0.003, Q 1 vs 2: 0.09, Q 1 vs 3: 0.09.

There was a strong relationship between HFD in the first year and subsequent transplant-free survival. Subjects in the lowest quartile of HFD (quartile 1) had worse long-term outcomes compared with those in the higher quartiles (Figure 2). Three- and 5-year post-transplant Kaplan-Meier transplant-free survival estimates in quartile 4 were 98% and 94%, respectively, compared with 79% and 56% in quartile 1 (*p* < 0.01). On univariate analysis, the hazard ratio for HFD in the first year for subsequent transplant-free survival was 0.990 (95% confidence interval [CI]: 0.985-0.994; *p* < 0.001). For every 10-day increase in HFD, the risk of death or retransplant decreased by 10%. After adjusting for age, type of transplant, ARS, and AMR, HFD remained a significant predictor of transplant-free survival (Table 4). Adjusting for AKI stage 3 and PGD grade 3 also did not appreciably change the effect of HFD. Both the initial transplant episode length of stay and number of readmission days were separately associated with transplant-free survival on univariate Cox regression analysis but lost significance after adjusting for HFD (data not shown).

**Table 4:** Association of Hospital-Free Days With Death or Retransplant

Per 10-day increase in hospital-free days (HFD)	Hazard ratio	95% CI	*p* value
Unadjusted	0.90	0.85, 0.94	<0.001
Adjusted for age, type of tx, ARS, AMR	0.92	0.87, 0.97	<0.001
Adjusted for age, type of tx, AKI-3, PGD-3	0.91	0.86, 0.96	<0.001

Abbreviations: AMR, antibody-mediated rejection; AKI-3: stage 3 acute kidney injury; ARS, acute rejection score; CI, confidence interval; PGD-3, grade 3 primary graft dysfunction; type of tx, single vs bilateral transplant.

Association between HFD in first year and time to death or retransplant tested with Cox proportional hazards model.

The univariate linear regression results between selected clinical variables and HFD during the first year are presented in Table 5. Severe AKI and PGD during the early postoperative period were each associated with more than 50 fewer HFDs. Higher LAS at transplant and diagnosis group B were also associated with fewer HFD.

**Table 5:** Univariate Linear Regression Association Between Selected Clinical Variables and Hospital-Free Days in the First Post-Transplant Year

Characteristic (N = 238)	Beta	95% CI	*p*
Age	0.05	−0.44, 0.54	0.84
Body mass index	−0.4	−1.4, 0.6	0.45
Primary diagnosis group			
A	Ref	—	
B	−31	−59, −3	**0.031**
C	0.4	−22, 22	0.97
D	−5	−16, 6	0.36
Lung allocation score at transplant	−0.5	−0.85, −0.15	**0.006**
Diabetes			
No	Ref	—	
Yes	−8	−22, 5	0.22
Cytomegalovirus status			
High risk	Ref	—	
Nonhigh risk	6.9	−5.2, 19	0.26
Primary graft dysfunction grade 3			
No	Ref	—	
Yes	−53	−71, −35	**<0.001**
Acute kidney injury stage 3			
No	Ref	—	
Yes	−59	−76, −41	**<0.001**
Acute rejection score	1	−17, 19	0.92
Antibody-mediated rejection			
No	Ref	—	
Yes	−16	−34, 1.5	0.07

Abbreviation: CI, confidence interval.

Bold indicates significant P value.

## Discussion

In this single-center experience, we have shown that HFDs in the first year after lung transplantation were a strong predictor of subsequent transplant-free survival, independent of several potentially confounding variables. While previous reports have analyzed the impact of transplant episode length of hospital stay and early readmissions on the long-term outcomes of lung transplant recipients, this is the first study to analyze the effect of a comprehensive measure of hospitalization during the critically important first postoperative year.

Two studies analyzed the impact of prolonged transplant episode LOS on survival in the United Network for Organ Sharing database roughly during the same era. Banga et al observed a 2-fold increased risk of 5-year mortality among recipients with an initial LOS over 25 days; however, this appears to have been mainly related to the strong effect on 1-year survival with a hazard ratio of 4.^2^ Another report found that recipients discharged after an LOS exceeding 90 days (>97.5 percentile) had 3 and 5-year survival of 26% and 16%, respectively.^3^ Several factors were identified as predictors of prolonged initial LOS, including the need for dialysis, postoperative stroke,^3^ and greater severity of illness before transplant.^2^ Neither study examined the effect of transplant episode LOS conditional upon 1-year survival.

Several single-center reports have assessed the characteristics of readmissions and their impact on outcomes.^18^ A large majority of recipients experience at least 1, and often multiple, readmissions during the first year,^4^, ^5^, ^6^, ^19^, ^20^ consistent with our proportion of 67% with a median of 2 admissions. Infection has been the most common cause of rehospitalizations in previous reports, similar to our experience. In a cohort of 367 1-year survivors at the University of Washington, each readmission during the first year was associated with a 22% increased risk of subsequent mortality^6^ and the University of Wisconsin group reported a 4.5-fold increased risk of death among recipients with over 4 readmissions in the first post-transplant year.^5^ Courtwright et al reported a mortality hazard ratio of 1.89 with any unplanned early (within 30 days of a discharge) rehospitalization in the first year.^4^ Several factors have been identified as risk factors for readmissions, such as airway complications,^6^ age, lower capacity to engage in self-care,^20^ male gender,^5^ and discharge to a long-term acute care hospital facility.^4^

Our findings extend these previous observations linking hospitalization in the first post-transplant year with long-term survival. HFD is a composite variable of hospital duration, including the initial transplant episode and readmissions, and thus may represent a more robust predictor of outcome. Fewer HFD during the first year remained a significant risk factor for subsequent mortality after adjusting for several covariates known to be associated with reduced survival and CLAD, including age, single lung transplant, cellular and AMR, AKI, and PGD.

The basis for the association between the duration of acute hospital stays during the first year and long-term survival is not clear based on our results and the existing literature. Early postoperative events such as severe PGD and AKI prolong initial LOS and are known to impact survival.^21^ While AKI stage 3 and PGD grade 3 influenced HFD in this study, the latter remained predictive of survival even after adjusting for these complications.

CLAD is the leading cause of long-term mortality and retransplant after lung transplantation^16^ and was the most common cause in our cohort. Repeated allograft injury from infections and other insults that prompt hospitalization could increase the risk of CLAD.^22^ More subtle factors can be invoked. Frailty at the time of discharge was a strong predictor of a subsequent unplanned hospitalization within 30 days^23^ and the presence of post-transplant frailty was recently shown to increase the risk of CLAD by 75%.^24^ Psychosocial variables could account for a greater need for hospitalization,^4^ while also affecting medical adherence and thereby long-term graft survival.^6^

Irrespective of the mechanisms accounting for the association between HFD and survival, our findings have potentially important implications. First, recognition that recipients with prolonged and repeated hospitalizations are at high risk could focus more attention and resources on identifying and addressing modifiable factors that may alter long-term outcomes. Second, if validated as a surrogate marker of long-term survival, HFD in the first year could serve as a readily obtainable and pragmatic end-point for interventional trials during the early post-transplant phase. Beyond its predictive ability for long-term survival, less time in the hospital, on its own, would be a favorable outcome for patients.^7^

The limitations of this study include the single-center nature and limited sample size, including number of events. Findings would need to be replicated in a larger, multicenter study. The impact of HFD was not adjusted for several variables that could have influenced survival such as pretransplant or post-transplant frailty, immunosuppression regimen, psychosocial factors, and others. Inpatient encounters from our institution were readily extractable from the electronic medical record, but we could not account for those occurring outside hospitals. However, these were considered to represent a negligible proportion of hospital days in the first year for this cohort as it has been our policy to direct patients to our facility when in need of admission and to routinely transfer those who are admitted elsewhere.

In summary, fewer HFDs during the first year after lung transplantation are associated with an increased risk of subsequent mortality or retransplant. Further research is warranted to better understand the basis for this observation and to determine if this index can be utilized as a surrogate end-point in clinical trials.

## Disclosure statement

The authors declare the following financial interests/personal relationships which may be considered as potential competing interests: Reda Girgis reports administrative support, article publishing charges, and statistical analysis were provided by Corewell Health West Michigan. The other authors declare that they have no known competing financial interests or personal relationships that could have appeared to influence the work reported in this paper.

This study was supported by institutional funding from the Frederick Meijer Heart and Vascular Institute. The authors are grateful to Mathew Lypka and Mathew Phad from the Scholarly Activity and Scientific Support services at Corewell Health for assistance with the statistical analysis.
